# Evaluating a virtual reality sensory room for adults with disabilities

**DOI:** 10.1038/s41598-022-26100-6

**Published:** 2023-01-10

**Authors:** Caroline J. Mills, Danielle Tracey, Ryan Kiddle, Robert Gorkin

**Affiliations:** 1 School of Health Sciences, Western Sydney University, Campbelltown Campus, Locked Bag 1797, Penrith, NSW 2751 Australia; 2 School of Education, Western Sydney University, Bankstown Campus, Locked Bag 1797, Penrith, NSW 2751 Australia; 3 Translational Health Research Institute, Western Sydney University, Locked Bag 1797, Penrith, NSW 2751 Australia; 4 Innovation Campus, Bunji Solutions, University of Wollongong, iAccelerate, Building 239Squires Way, North Wollongong, NSW 2500 Australia; 5 SMART Intrastructure Facility, University of Wollongong, Wollongong, NSW 2522 Australia

**Keywords:** Psychology, Health care, Engineering

## Abstract

Sensory processing difficulties can negatively impact wellbeing in adults with disabilities. A range of interventions to address sensory difficulties have been explored and virtual reality (VR) technology may offer a promising avenue for the provision of sensory interventions. In this study, preliminary evidence about the impact of Evenness, an immersive VR sensory room experience, for people with disabilities was investigated via a single intervention pre-post mixed methods design. Quantitative methodology included single intervention pre-post design (five month timeframe) with 31 adults with various developmental disabilities to determine the impact of use of aVR sensory room using a head mounted display (HMD) in relation to anxiety, depression, sensory processing, personal wellbeing and adaptive behaviour. Qualitative semi-structured interviews were also conducted with thirteen purposefully selected stakeholders following Evenness use. Results indicated significant improvements in anxiety, depression and sensory processing following Evenness use. Qualitative analysis corroborated the anxiety findings. No significant changes were observed in personal wellbeing or adaptive behaviour. Results are promising and indicate that a VR sensory room may have a positive impact on anxiety, depression and sensory processing for adults with disabilities. A longer study timeframe and a more rigorous experimental methodology is needed to confirm these findings.

## Introduction

Sensory processing difficulties are often prevalent in people with intellectual disabilities^1^ and autism^2^. Deficits in sensory processing impact the process of taking in and organising information from the senses, then interpreting and using information in a functional way to support participation in daily activities^3^ Under Dunn’s model of sensory processing these difficulties range from *under-responsive* where a person registers sensory input later than others to *overresponsive* individuals who register sensory input sooner and more intensely^4^.

Although not recognised as a specific disorder, sensory processing issues are linked to poor physical and mental health^5,6^. Sensory processing difficulties can result in limitations with self-care^7^, participation in the community^8^, quality of life^9^ and adaptive functioning^10^. Further, sensory processing difficulties and anxiety were observed to co-occur in children with ADHD^5^ and autistic children^11^. Depression and sensory processing difficulties have also been linked in adults with affective disorders^12^.

Despite known difficulties and associated negative impacts, there is little agreement regarding suitable interventions to support sensory difficulties in people with disabilities^13^. Studies lack clarity and methodological rigour to identify the most effective intervention types, dosing and outcomes targeted^13,14^ and where studies investigating sensory interventions in adult populations do occur, they largely focus on mental health settings^15,16^.

Whilst cognizant of limitations, emerging evidence supports the use of a targeted ‘sensory diet’ for improving sensory processing in children^17^. Similarly, the use of targeted sensory activities through a sensory activity schedule points to benefits across a range of outcomes, including classroom task performance^18,19^ and cognitive strategy use^20^ for autistic children with intellectual disability. Studies were small and preliminary in nature with authors highlighting the individual nature of sensory interventions. Improvements in cognitive strategy may indicate that sensory-based interventions could potentially have utility in addressing problems which are not strictly sensory in nature^20^.

Sensory interventions utilising a sensory room are common within the research and practice^21–23^ with rooms including visual stimuli like bubble tubes and projected visual effects^24^, auditory input such as nature sounds or dimmed music, and tactile input such as vibrating massagers^25^. Sensory rooms have been implemented in environments ranging from schools to psychiatric facilities for decades^21^ often with the purpose of self-managing distress, assisting with emotional regulation^15^ or managing behaviour^22^.

A meta-analysis conducted by Lotan and Gold^22^ reported improvements in adaptive functioning, maladaptive behaviour and daily interactions following sensory room use. Included studies encompassed people of a wide range of ages (5–65 years) with most participants having moderate to severe intellectual disability, some with co-occurring autism. Similarly, a study by Shapiro and colleagues found that a physical sensory room (termed ‘sensory adapted environment’) reduced anxiety in children with and without developmental disability (n = 32, mean age 8) by utilising lighting, tactile, auditory and somato-sensory stimulation during dental treatments^26^. In addition, a recent scoping review by Breslin, Guerra^21^ noted improvements in behaviour, alertness and anxiety following use of a sensory room using a range of measures including that of distress, discomfort and maladaptive behaviours (e.g., aggression, stereotypy, self-injury), as well as positive behaviours (e.g., communication, engagement, cooperation). Authors recommended sensory room use be further explored exclusively in people with mild to moderate disability, particularly autism and intellectual disability^21^. In contrast, a scoping review by Cameron, Burns^23^ on the use of multi-sensory environments (sensory rooms) with children and adults with intellectual and developmental disabilities found the overall evidence to be promising but inconclusive due to large variations in intervention type, frequency and duration of use.

While there is some consensus in the literature of the perceived benefit of traditional sensory rooms, requirements of physical spaces present logistical issues ^27^. This potentially limits access for a range of users, particularly people with disability who may have limited access to required resources ^27^. Literature has also highlighted the importance of further research into innovative sensory interventions which fit into a person’s own context ^20^.

Immersive virtual reality (VR) offers an emerging solution to create effective interventions and experiences with enhanced accessibility^28^. VR is a uniquely suited portable technology, whereby developers can design, and users can experience, a vast ecosystem of simulated, immersive, personalised computer-generated stimuli that can impact the major senses of vision and hearing^29^. Recent VR experiences are also utilising haptic feedback through vibrating hand controllers in order to simulate real life touch experiences^30^. In comparison to physical rooms, VR sensory rooms overcome infrastructure limitations of needing a dedicated isolated area, with the associated costs and maintenance. Potential drawbacks have been noted with VR usage including implementation safety and control when using VR with head-mounted displays (HMD)^28,31^. Studies have documented the potential negative impacts of cybersickness on a range of users including autistic users (children and adults)^31^ and older and younger adult users without disabilities^32^. In addition, problems with using VR with a HMD have been widely noted for adults with and without disabilities^31,32^ .Despite these potential difficulties, VR may offer greater accessibility for users through simple on-demand setup, portable equipment, and allowing greater throughput by adding more components.

VR has already shown promising findings for people with disabilities to enhance functional communication^28,33^, daily living skills^34^ and leisure participation^35^. Only a single study was identified which targeted VR sensory intervention for people with disabilities. Wuang, Chiang ^36^ found that children aged 7 to 12 with Down syndrome who utilised a Wii-based VR sensory intervention twice a week for 24 weeks had better outcomes in motor skills and sensory integration than children who received occupational therapy intervention as usual. No studies were located where a VR sensory intervention was utilised with adults with disabilities or measured impact on wellbeing or adaptive behaviour.

Noting potential benefits, our team explored a new offering to provide calming or stimulating sensory input aiming to enhance sensory processing, wellbeing, and adaptive behaviour of people with disability through the VR program, Evenness (Devika, Wollongong, Australia). This virtual analogue is one of the world’s first representations of the commonly used physical sensory room and offers sensory processing interventions in a virtual space. The present study identifies reported benefits of the Evenness VR Sensory Room and the impact according to the user’s age, frequency of use, disability diagnosis and initial needs. We also explore benefits from both user, carer and staff perspective.

## Materials and methods

### Design

The preliminary pilot study adopted a single intervention pre-post mixed method design^37^ where quantitative data was collected at both pre and post-test via survey, while qualitative data was completed at post-test only via interview. In this study, the primary research aim, and therefore methodology, was quantitative in nature and qualitative findings served as a means of clarification and interpretation of quantitative data^38^. For this reason, only a small number of qualitative interviews were conducted. There was an average of 157 days (5 months) between pre and post data collection (SD = 17) ranging between 133 and 197 days. The role of the length and duration of a VR intervention on outcomes is under-researched^39^ resulting in little guidance for researchers and practitioners. A recent systematic review of VR social skill intervention for adults with intellectual disability identified that interventions lasted between 20 weeks and 18 months, and 1 to 40 sessions, however, duration was not reported^40^. Where the impact of duration has been investigated, recent research, Villena-Taranilla and colleagues^41^ suggest that short interventions (less than two hours) are more effective.

### Participants

**Figure 1:**
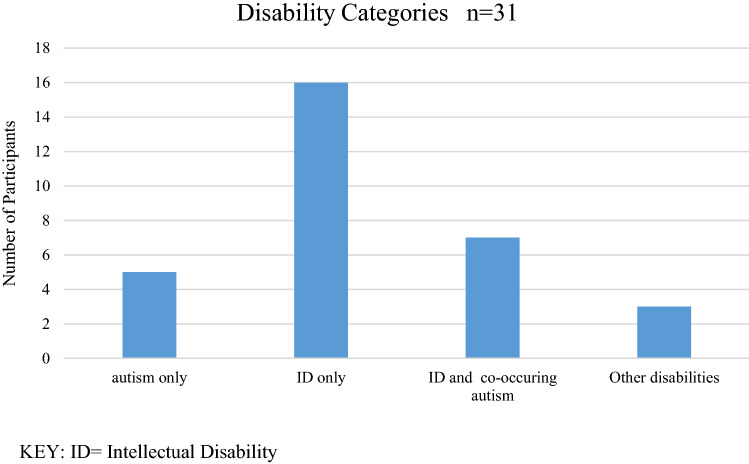
Participant disability categories.

Participants included 31 adults with disabilities where 16 (50.0%) were diagnosed with intellectual disability (ID) but not autism, 8 (25.0%) were diagnosed with both ID and autism, and 4 (12.5%) were diagnosed with ASD but not ID[see Fig. 1]. Diagnoses were provided to the researchers upon enrolment in the study. At pre-test, 20 (64.5%) of respondents were aged less than 30 years, while the remaining 11 (35.5%) were aged 30 years or more. Participants were grouped into four categories for statistical analysis. The ‘autism’ group (n = 12) is comprised of those with autism only (n = 4) and those with autism and co-occurring ID (n = 7). The ‘ID’ group (n = 23) is comprised of those who have ID only (n = 16) and those with autism and ID (n = 7). Participants used Evenness on average 6 times (range 2–8) over the 5 month study period. The average length of total usage during this period was 26 min and 7 s with a median time use of 13 min and 54 s (range 0 min to 2 h, 38 min and 53 s). Average use in one instance was 4 min and 32 s with single uses ranging from a few seconds to 45 min. Participants were able to determine their own usage and there was no set duration. Potential participants were excluded if they had any medical condition which would preclude safe use of a VR sensory room, including epilepsy or other seizure condition. Potential participants were asked to discuss this with their carer and their own medical practitioner before consenting in order to ensure safe participation.

Upon commencement, participants reported the following sensory profiles as measured by the Adolescent Adult Sensory Profile (AASP)^42^. For three of the four quadrants, at least half of the participants were ‘similar to most’ at pre-test (low registration, n = 16; sensation seeking n = 18 and sensory sensitivity n = 19). This indicates that according to the AASP, most participants did not present with sensory processing difficulties for these three quadrants at pre-test. For sensation avoiding, 10 were similar to most at pre-test, with 21 participants scoring more or less than most. Three quarters of participants were outside of normal range for sensation avoiding at pre-test, indicating sensory processing difficulties in this area.

Qualitative interviews were conducted with a purposefully selected sample of five of the 31 participants with disabilities who were representative of the sample in terms of age, disability, pre-test scores and VR usage. Participants ranged from 20 to 61 years old with a range of different disabilities including autism and co-occurring intellectual disability, cerebral palsy with co-occurring intellectual disability, moderate intellectual disability and Klinefeher syndrome. All attended the interviews with their carers. In addition, three staff members were interviewed who were experienced in providing support for people with disability and responsible for day-to-day implementation of the VR Sensory Room.

### Procedure

This study was conducted with ethical approval from Western Sydney University’s Human Research Ethics Committee (HREC) and was conducted in line with ethical procedures. This HREC is constituted and operates in accordance with the National Statement on Ethical Conduct in Human Research 2007 (Updated 2018). Approval granted 1st June 2020, No: H13815. Informed consent was guided by the needs of the individual participant following ethical procedures.

Participants were recruited from the host organisation through purposive and convenience sampling. They were over 18, had a diagnosed disability and were able to attend at least two VR sessions over the course of the study. Participants were recruited via flyers and verbal information made available through their affiliation with the disability organisation which hosted the Evenness experience at no cost to participants. Interested participants and carers were provided with further information about the study. If they opted to participate, they gave written informed consent for their participation in the study, with the support of their carer if needed. Carers of participants and staff members involved in supporting Evenness usage were also included as participants and gave written informed consent for their own participation. VR users were excluded if they had a medical or health condition which would preclude safe use of VR. They were asked to discuss their own circumstances with their carers and appropriate health professionals prior to taking part.

Evenness was designed for immersive VR, specifically using the head-mounted VR unit with six degrees of freedom and multi-sensor inputs. To prepare the experience, the Evenness VR Sensory Room was installed on a Windows 10 computer with a VR-ready graphics card, a HP Reverb G2 headset unit, and two Windows Mixed reality hand controllers. The staff would set up the space by starting the SteamVR & Mixed Reality portal and trace out the physical areas for the VR headset, followed by logging on to the Evenness application. The user would then put on the headset and freely engage with the experience via both the headset and controllers.

**Figure 2:**
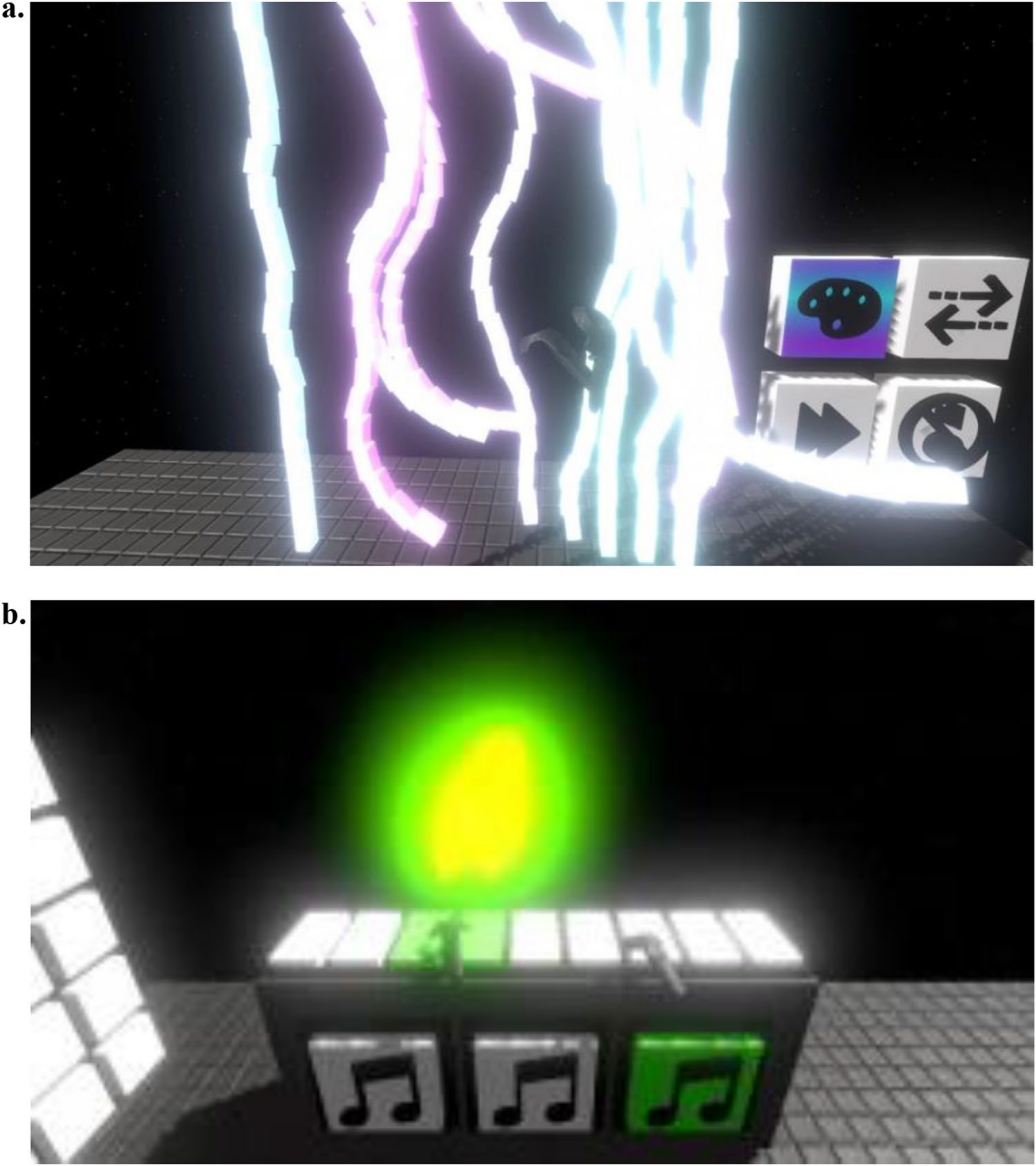
Evenness VR Sensory Room: Screen shots of interactive spaces from the user perspective. (**a**) light curtain with colour change option and (**b**) interactive piano).

The Evenness Sensory Room was designed by Devika to replicate a physical sensory room where users can freely engage with items on their own agenda and timeline. Evenness replicates previously used aspects of traditional practice where physical sensory rooms used by people with disabilities contain a range of sensory experiences including auditory (music), visual (interactive light panels, light curtain, lava lamp) and touch experiences (vibration and massage)^1,25^. Figure 2 shows screenshots of the Evenness VR experience from the user’s perspective.

Participants were able to access Evenness as part of their participation in the organisation’s day program at their request. Some participants attended the day program where the VR equipment was located and others who attended surrounding day programs were able to book Evenness use into their weekly activities. Participants were provided with transportation to access Evenness. Each participant using Evenness had their nominated participating carer with them throughout their use. The carer was a staff member of the organisation or their family member, was known and trusted by the participant and was trained and experienced in operating the VR equipment. VR technical support was also provided. Evenness usage was recorded using a time log noting the frequency (amount of instances of use during the study period) and duration of VR usage (total minutes of usage across all instances of use during the study) for each participant.

### Instruments

This research utilised five quantitative measures described below in addition to qualitative semi-structured interviews.

#### GAS-ID

The Glasgow Anxiety Scale for people with an intellectual disability^43^ comprises three subscales including worries, specific fears, and physiological symptoms respectively. 27 items were administered and a Likert-type response scale presented (three response options; scored 0–2 where some items are reversed scored). The maximum scores are: worries 20; specific fears 18; physiological symptoms 16; total 54, where higher scores indicate higher symptoms of anxiety. Mindham and Espie ^43^ suggest that a score of 13 be used to identify a possible anxiety disorder.

#### GDS-LD

The Glasgow Depression Scale for people with a learning disability^44^ is not presented as a diagnostic measure but rather a depressive-symptom rating. 20 items were administered and a Likert-type response scale presented (three response options; scored 0–2 where some items are reversed scored). The maximum total score possible is 40 where higher scores indicate higher symptoms of depression. Cuthill et al.^44^ suggest a score of 13 provides an indicator of depression.

#### AASP

The Adolescent Adult Sensory Profile is a 60-item standardised self-rated questionnaire which was developed to understand sensory processing patterns in adolescents and adults ^42^. On a five-point Likert rating scale, participants identify how often they respond to a particular sensory stimulus from almost never to almost always. The questionnaire is divided into six sections including Taste/Smell Processing, Movement Processing, Visual Processing, Touch Processing, Activity Level and Auditory Processing. Responses are scored according to the four sensory processing quadrants from Dunn’s model of sensory modulation ^4^ including *Low Registration and Sensation Seeking (characterised as hyporesponsive sensory styles) and Sensory Sensitivity* and *Sensation Avoiding (hyperresponsive sensory styles)*. Each of the four quadrant scores are classified into three broad categories ranging from less than others, similar to others and more than others. These scores are intended to categorise responses with regard to how they compare statistically to the general population. A score in the ‘less than others range (comprising ‘much less’ or ‘less than others’) indicates less than 16% of the population fell into that category (more than one standard deviation from the mean). A score of similar to others falls within one standard deviation of the mean (16–84%) and a score in the more than others range (comprising ‘much more’ or ‘more than others’) indicates a score greater than 84% of the population (more than one standard deviation above the mean)^42^.

#### PWI

The Personal Wellbeing Index– Intellectual Disability 3rd Edition^45^ comprises 8 single-item scales where participants rate how satisfied they are with various aspects of their life on a 11-point Likert-type response scale (0 very sad − 10 very happy). Prior to the administration of the substantive questions, competence in understanding the response scale is assessed. Six participants failed this trial and were unable to continue with the support of a carer, hence the results are based on the remaining 25 participants.

#### ABAS-3

Adaptive Behaviour Assessment System is a norm referenced standardised questionnaire which is rated by the person or a caregiver^46^. It measures a person's adaptive skills in 10 areas including communication, community use, functional academics, home living, health and safety, leisure, self-care, self-direction, social and work. The scale is rated on a 4-point scale from 0 to 3. A score of 0 denotes the person has not acquired the skill to an age-appropriate level while 1 denotes a performance deficit in the skill. A score of 2 indicates more skilled and 3 indicates the person has the required skills. Scores are tallied for each adaptive skill area and converted to scaled scores which give an indication of the person's adaptive skills in comparison to others in the same age group. A scaled score of 8–10 indicates the person scored within the average range for that age group.

#### Semi-structured qualitative interviews

Semi-structured interview schedules were designed to gather the perspectives of participants, their carers and staff about the use of Evenness for individual participants as well as perspectives of benefits or drawbacks. Staff gave their perspectives on what they observed in their role coordinating use of Evenness in relation to all participants. Interview guides varied slightly for the three participant groups, with simplified questions being offered to participants with disabilities. Semi-structured interviews with participants with disabilities were conducted by a researcher who was trained and experienced in supporting people with disabilities. Interviews ranged from 4 to 40 min (mean: 15 min).

### Data analysis

#### Quantitative analysis

First, exploratory data analysis was conducted using a bar plot and Shapiro–Wilk test statistics to check for assumption of normality. For participants with missing data, five imputations were performed to replace missing values using the ‘zoo’ package in the R environment. Imputation procedures have been used in prior studies and are recommended in methodological literature^47^.

Second, a Wilcoxon signed ranks test was employed to calculate the change between pre-test and post-test scores for each outcome measure. For this study, a Wilcoxon signed ranks test was selected because of the small sample size, the use of outcome measures that contained ordinal data and the need for a test which did not assume a normal distribution^48^, In addition, the Wilcoxon signed ranks test relies on the W-statistic, and this approximates a normal distribution for samples with n > 10 paired observations. Sample size was calculated using G*Power software and based on the parameters used in dependent samples testing (Wilcoxon) with an effect size (r) of 0.6 (large). In order to yield 80% power with an alpha value of 0.05, a minimum sample of 23 was determined to be appropriate. Recruitment was targeted at a higher number (31) to account for possible participant withdrawal during the study.

Last, Spearman’s correlation was used to examine the relationship between duration of use of the Evenness VR sensory room and the mean differences for the study outcomes between pre-post periods. Spearman’s correlation was selected as it allows evaluation of a monotonic relationship between two continuous or ordinal variables and most of the outcomes in the current study have a non-linear relationship ^49^.

### Qualitative analysis

Semi-structured interviews were recorded and transcribed verbatim and these transcripts formed the data set for analysis. Reflexive thematic analysis of transcripts was utilised to achieve a comprehensive understanding of the data^50^ with the aim of understanding the implicit meaning, or essence, of participants’ experiences and to identify the most pertinent ideas related to the research objectives. Six stages of reflexive thematic analysis as described by Braun and Clarke^50^ were used. First, transcripts were read by two independent researchers for the purposes of familiarisation and initial code generation. Both researchers had clinical backgrounds (occupational therapy and psychology) and had significant experience supporting people with disabilities. Codes were then reviewed and conceptually similar codes were grouped together. Coding was reviewed and initial themes and sub-themes were developed in relation to the research objectives. Following this process, the two researchers met to ‘triangulate’ and finalise themes in light of research objectives before commencing the collaborative process of writing up the qualitative findings. Trustworthiness as originally defined by Lincoln and Guba^51^ was established through the process of triangulation by two researchers who were experienced in the field of disability research.

## Results

An analysis of the pre-post quantitative data and post qualitative data addresses the key objective of the study to identify the reported benefits of Evenness for participants according to age, disability, usage or initial needs. Tables 1 and 2 present the results of the quantitative analysis while themes identified via the qualitative analysis appear according to the outcome constructs of interest.

**Table 1:** Pre-post statistical analysis of depression (GDS-LD), and anxiety (GAS-ID) with Wilcoxon Signed Ranks test.

Study variables	n	Pre-test		Post-test		Pre-Post difference		Wilcoxon signed ranks test
		Mean (± SD)	Shapiro–Wilk test (*P* value)	Mean (± SD)	Shapiro–Wilk test (*P* value)	Mean (± SD)	Shapiro–Wilk test (*P* value)	*P* value
***Glasgow Depression Scale (GDS-LD)***								
Overall	31	11.87 (4.86)	0.827	9.97 (5.04)	0.076	− 1.90 (6.70)	0.300	0.079
Depressed at pre-test	14	16.00 (3.35)	0.014	9.86 (4.80)	0.177	− 6.14 (5.50)	0.087	0.002*
Not depressed at pre-test	17	8.47 (2.83)	0.032	10.06 (5.37)	0.235	1.59 (5.57)	0.234	0.299
ID group	23	12.09 (5.12)	0.861	10.04 (4.49)	0.500	− 2.04 (7.22)	0.486	0.147
Autism group	12	10.75 (4.43)	0.349	11.58 (4.66)	0.293	0.83 (5.62)	0.075	0.964
< 30 years	20	10.85 (4.56)	0.017	9.70 (5.04)	0.465	− 1.15 (6.38)	0.208	0.300
≥ 30 years	11	10.45 (5.24)	0.397	13.73 (5.06)	0.203	− 3.27 (7.36)	0.405	0.123
**Glasgow Anxiety Scale (GAS-ID)**								
*Overall anxiety*								
Overall	31	20.00 (9.10)	0.293	14.19 (9.05)	0.153	− 6.81(9.59)	0.426	< 0.001*
Anxiety at pre-test	28	22.46 (8.29)	0.013*	15.07 (9.06)	0.423	− 7.39 (9.86)	0.402	< 0.001*
No anxiety at pre-test	3	7.33 (2.08)	0.463	6.00 (2.65)	0.363	− 1.33 (4.04)	0.726	0.750
ID group	23	21.65 (9.30)	0.709	14.35 (8.91)	0.171	− 7.30 (10.75)	0.775	0.006*
Autism group	12	22.00 (10.66)	0.008*	16.25 (7.53)	0.832	− 5.75 (9.62)	0.054	0.025*
< 30 years	20	18.85 (9.08)	0.305	11.60 (5.76)	0.745	− 7.25 (8.81)	0.144	0.002*
≥ 30 years	11	24.91 (8.10)	0.956	18.91 (12.02)	0.966	− 6.00 (11.29)	0.733	0.119
*Worries*								
Overall	31	5.77 (3.63)	0.794	4.32 (3.49)	0.624	− 1.45 (4.04)	0.785	0.055
ID group	23	7.96 (3.39)	0.963	6.13 (3.71)	0.359	− 1.83 (4.27)	0.800	0.062
Autism group	12	8.75 (2.22)	0.395	7.92 (3.37)	0.902	− 0.83 (3.07)	0.036	0.401
< 30 years	20	7.35 (2.98)	0.171	5.20 (3.29)	0.793	− 2.78 (2.56)	0.480	0.015*
≥ 30 years	11	9.82 (3.28)	0.958	8.18 (2.56)	0.666	− 2.15 (3.45)	0.212	0.474
*Specific fears*								
Overall	31	5.62 (3.55)	0.181	4.31 (3.61)	0.035	− 1.31 (4.04)	0.419	0.094
ID group	23	6.22 (3.11)	0.403	4.65 (3.31)	0.058	− 1.56 (4.35)	0.341	0.878
Autism group	12	4.92 (4.50)	0.008	4.75 (3.11)	0.912	− 0.17 (4.13)	0.434	0.766
< 30 years	20	4.55 (3.37)	0.041	3.70 (2.36)	0.155	− 0.85 (3.88)	0.096	0.230
≥ 30 years	11	8.00 (3.06)	0.730	5.45 (4.87)	0.151	− 2.54 (4.27)	0.967	0.092
*Physiological symptoms*								
Overall	31	6.84 (5.28)	< 0.001*	3.58 (3.43)	< 0.001*	− 3.26 (5.38)	< 0.001*	0.001*
ID group	21	7.34 (5.81)	< 0.001*	3.52 (3.70)	< 0.001*	− 3.83 (5.85)	0.004*	0.003*
Autism group	11	8.08 (7.35)	0.001*	3.58 (2.50)	0.657	− 4.50 (7.23)	0.008*	0.030*
< 30 years	20	6.70 (6.20)	< 0.001*	2.65 (1.98)	0.105	− 4.05 (5.92)	0.001*	0.003*
≥ 30 years	11	7.09 (3.24)	0.207	5.27 (4.80)	0.335	-1.82 )4.09)	0.687	0.141

Shapiro–Wilk was used for normality check.

Asterisk indicates statistical significance.

**Table 2:** Pre-post statistical analysis of sensory processing (AASP) with Wilcoxon Signed Ranks Test.

Study variables	n	Pre-test		Post-test		Pre-Post difference		Wilcoxon signed ranks test
		Mean (± SD)	Shapiro–Wilk test (*P *value)	Mean (± SD)	Shapiro–Wilk test (*P* value)	Mean (± SD)	Shapiro–Wilk test (*P* value)	*P* value
**Adolescent Adult Sensory Profile (AASP)**								
*Low registration*								
Overall	31	36.04 (9.38)	0.026*	41.42 (10.11)	0.283	5.35 (11.14)	0.529	0.006*
Similar to most at pre test	16	30.31(3.42)	0.218	40.31(10.73)	0.689	10.00(9.77)	0.923	0.002*
More than most at pre test	13	45.15(6.594	0.108	44.62(8.69)	0.431	0.54 (9.85)	0.423	0.807
Less than most at pre test	2	23 (0)	–	29.5(0.71)	–	6.50(0.71)	–	0.180
ID group	23	36.69 (9.46)	0.033*	42.09 (10.68)	0.520	5.39 (11.94)	0.737	0.023*
Autism group	12	33.92 (8.26)	0.625	40.50 (10.75)	0.508	6.58 (10.04)	0.608	0.099
< 30 years	20	32.55 (6.79)	0.216	39.35 (9.66)	0.443	6.80 (12.39)	0.452	0.015*
≥ 30 years	11	42.45 (10.34)	0.048	45.18 (10.25)	0.481	2.72 (8.28)	0.999	0.284
*Sensation seeking*								
Overall	31	45.03 (9.31)	0.858	42.26 (8.06)	0.406	-3.77 (9.78)	0.918	0.056
Similar to most at pre test	18	49.28(4.28)	0.208	41.72(7.54)	0.165	7.56 (9.87)	0.256	0.006*
More than most at pre test	3	62.00 (3.00)	–	39.00 (2.65)	–	23.00 (5.57)	–	0.109
Less than most at pre test	10	35.40(4.58)	0.184	44.20(9.56)	0.992	8.80 (12.25)	0.698	0.041*
ID group	23	47.56 (9.27)	0.859	41.17 (8.24)	0.221	-6.39 (9.69)	0.606	0.007*
Autism group	12	45.42 (7.59)	0.879	41.36 (7.89)	0.543	-3.36 (10.65)	0.301	0.239
< 30 years	20	47.35 (7.74)	0.775	41.40 (7.87)	0.166	-5.95 (9.41)	0.580	0.023*
≥ 30 years	11	43.64 (11.69)	0.380	43.82 (8.12)	0.666	0.18 (9.59)	0.582	0.999
*Sensory sensitivity*								
Overall	31	37.09 (9.17)	0.030*	38.77 (11.54)	0.171	1.68 (10.58)	0.605	0.335
Similar to most at pre test	19	33.26(4.52)	0.538	36.63(10.60)	0.133	3.37(10.91)	0.269	0.204
More than most at pre test	10	47.00(7.82)	< 0.001*	42.50 (13.00)	0.276	4.50(10.70)	0.435	0.168
Less than most at pre test	2	24.00(1.41)	–	40.50 (14.85)	–	16.50(16.26)	–	0.180
ID group	23	37.43 (9.45)	0.014*	39.52 (10.58)	0.420	2.09 (11.12)	0.795	0.394
Autism group	12	33.92 (7.49)	0.698	42.75 (11.47)	0.608	8.83 (7.35)	0.444	0.004*
< 30 years	20	32.80 (5.83)	0.620	37.55 (9.86)	0.170	4.75 (9.38)	0.992	0.036*
≥ 30 years	11	44.91 (9.15)	0.046	41.00 (14.35)	0.395	-3.91 (10.75)	0.530	0.261
*Sensation avoiding*								
Overall	31	41.06 (11.06)	0.242	40.81 (11.78)	0.955	-0.26 (12.12)	0.896	0.922
Similar to most at pre test	10	32.00(2.40)	0.380	40.00(10.86)	0.445	8.00(10.54)	0.381	0.032*
More than most at pre test	18	49.28(5.36)	0.027*	42.50 (12.44)	0.491	6.78 (12.65)	0.121	0.029*
Less than most at pre test	3	22.00(1.73)	–	33.33(11.15)	–	11.33 (10.69)	–	0.109
ID group	23	41.87 (10.49)	0.185	42.09 (9.53)	0.629	0.22 (11.83)	0.981	0.889
Autism group	12	38.75 (11.65)	0.587	43.42 (12.74)	0.165	4.67 (10.86)	0.687	0.272
< 30 years	20	36.65 (10.08)	0.216	39.50 (10.37)	0.608	2.85 (12.18)	0.575	0.286
≥ 30 years	11	49.09 (8.02)	0.587	43.18 (14.222)	0.838	-5.09 (10.23)	0.974	0.092

Asterisk indicates statistical significance.

### Improvements in anxiety

Results showed a significant pre-test to post-test decrease in the overall mean anxiety (*p* < 0.001). A similar finding was observed among respondents who had anxiety at pre-test (score on the GAS-ID of 13 or above) (*p* < 0.001), had an intellectual disability (*p* = 0.006), had autism (*p* = 0.025) and were aged < 30 years (*p* = 0.002) (See Table 1). No significant correlation was observed between the duration of time spent in the VR Sensory Room and the mean difference in the reported anxiety between post- and pre-tests (*p* = 0.77) (see Table 4).

Pre- to post-test decreases were most evident in the Physiological Symptoms subscale where significant decreases were observed for the overall group (*p* = 0.001), those with intellectual disability (*p* = 0.003), autism (*p* = 0.030) and those aged less than 30 years (*p* = 0.003). For the Worries subscale, significant mean differences were observed for the overall group (*p* = 0.002), and among those aged < 30 years (*p* = 015). However, there were no significant differences in the Specific Fears subscale (see Table 1).

Qualitative analysis demonstrated that a reduction in anxiety was cast as the most prominent benefit to users. Examples include comments from a staff and user:

*‘The main one that we see is it helps with anxiety. So a lot of participants come in and they're quite heightened. They're having behaviours of concern, self-injurious behaviours.’ (Staff #2).*

*‘Just breathing in my own space, not worrying. It's hard to explain but, when I was in here, all my problems just went. I was just concentrating on what was around me more and the sounds and that was different… I can't wait for the next one actually. When I go to bed now and I have a few bad moments, I just look up and I just see the stars and that and I just – it’s changed me a lot actually.’ (61 year old participant with intellectual disability).*

### Improvements in depression

Results showed a significant reduction in the mean score for depression between the pre-test and post-test period among study participants who started the intervention depressed (GDS-LD score of 13 or above) (*p* = 0.002). However, no significant pre- and post-test differences were observed in depression scores across all study participants (*p* = 0.079), and among those who did not have depression at pre-test (*p* = 0.299), those aged < 30 years (*p* = 0.300), and those aged ≥ 30 years (*p* = 0.123) (see Table 1). No significant correlations were observed between the duration of time spent in the VR Sensory Room and the mean difference in the reported depression between post- and pre-tests as shown in Table 4 and in the qualitative.

### Change in sensory processing profiles

For low registration on the AASP, significant increases were observed between pre-test and post-test in participants overall (*p* = 0.006), participants with ID (*p* = 0.023), and those aged less than 30 years (*p* = 0.015). Significant increases in low registration were observed in those who were ‘similar to most’ at pre-test (*p* = 0.002). For sensation seeking, significant decreases were observed in those who were ‘similar to most’ at pre-test (*p* = 0.006), while significant increases were observed in those who were ‘less than most’ at pre-test (*p* = 0.041). Significant decreases from pre-test to post-test were observed for the ID group (*p* = 0.007) and those aged < 30 years (*p* = 0.023) [see Table 2].

Significant score increases were observed in sensory sensitivity for the autism group (*p* = 0.004) and for those aged less than 30 years (*p* = 0.036). However, there were no significant differences from pre-test to post-test observed in any other subgroups[see Table 2]. For sensation avoiding, significant score increases were observed in participants who were similar to most at pre-test (from a mean of 32 to a mean of 40, *p* = 0.032), however the mean score remained within the ‘similar to most’ range on the AASP. For those who scored more than most at pre-test, a significant decrease in sensation avoiding was observed (*p* = 0.029). No significant differences were observed in other sub-groups as shown in Table 2.

Spearman’s correlation analysis showed significant positive relationships between time spent in the VR Sensory Room and increased scores in low registration and sensory sensitivity as shown in Table 4.

### No significant change in personal wellbeing

Following use of the VR Sensory Room, some improvements were observed in satisfaction related to health (Diff = 4.00; SD = 17.79), personal safety (Diff = 4.74; SD = 21.95), and future security (Diff = 4.40; SD = 26.47). However, no improvements were found in the overall satisfaction (Diff = − 5.60; SD = 35.01), personal relationship (Diff = − 8.00; SD = 22.73), and community connectedness (Diff = − 2.00; SD = 26.93) and no statistically significant improvements were observed for the overall personal wellbeing index scale and all other subscales from pre-test to post-test (see Table 3). Furthermore, no significant correlation was observed between the duration of time spent in the VR Sensory Room and the mean difference in personal wellbeing between post-test- and pre-test (r = − 0.21, *p* = 0.32) as shown in Table 4. In the qualitative interviews, impact on satisfaction with these life domains was not identified as a theme.

**Table 3:** Pre-post statistical analysis of Personal Well-Being (PWI) and Adaptive Behaviour and Assessment System (3) (ABAS-3) with Wilcoxon Signed Ranks test.

Study variables	n	Pre-test		Post-test		Pre-Post difference		Wilcoxon signed ranks test
		Mean (± SD)	Shapiro–Wilk test (*P *value)	Mean (± SD)	Shapiro–Wilk test (*P *value)	Mean (± SD)	Shapiro–Wilk test (*P *value)	*P* value
**Personal Wellbeing Index**								
*Overall satisfaction (part I)*								
Overall	25	77.60 (23.89)	< 0.001*	72.00 (26.00)	< 0.001*	− 5.60 (35.01)	0.059	0.330
ID group	19	74.74 (18.29)	< 0.001*	66.84 (27.90)	0.002*	− 7.89 (38.95)	0.085	0.256
Autism group	11	86.00 (9.66)	< 0.001*	79.00 (12.87)	0.011*	− 7.00 (21.68)	0154	0.865
< 30 years	16	80.62 (20.81)	< 0.001*	75.00 (23.66)	0.001*	− 5.62 (32.24)	0.127	0.444
≥ 30 years	9	72.22 (23.86)	0.066	66.67 (31.22)	0.164	− 5.56 (41.57)	0.867	0.623
*Overall satisfaction (part II)*								
Overall	25	80.17 (12.07)	0.148	81.43 (14.05)	0.020*	1.26 (14.13)	0.053	0.310
ID group	19	78.72 (13.40)	0.028	78.27 (14.30)	0.263	− 0.45 (15.34)	0.172	0.687
Autism group	11	80.52 (11.97)	0.131	83.25 (11.90)	0.092	2.73 (15.78)	0.101	0.266
< 30 years	16	81.70 (11.25)	0.090	83.04 (12.70)	0.078	1.34 (15.38)	0.021*	0.268
≥ 30 years	9	77.46 (13.66)	0.754	78.57 (16.58)	0.625	1.11 (12.4&)	0.625	0.812
**Adaptive Behaviour and Assessment System (3) (ABAS-3)**								
*General adaptive composite*								
Overall	31	16.58 (13.61)	< 0.001*	18.45 (16.71)	< 0.001*	1.87 (12.39)	< 0.001*	0.897
ID group	23	15.22 (12.79)	< 0.001*	16.09 (13.75)	< 0.001*	0.87 (12.39)	< 0.001*	0.652
Autism group	12	14.00 (9.87)	< 0.001*	17.67 (19.92)	< 0.001*	3.67 (10.43)	< 0.001*	0.672
< 30 years	20	14.95 (11.37)	< 0.001*	13.30 (10.62)	< 0.001*	− 1.65 (4.90)	0.001*	0.168
≥ 30 years	11	27.82 (21.78)	0.112	19.54 (17.16)	0.022*	8.27 (18.58)	0.156	0.221

Shapiro–Wilk was used for normality check.

*ID* intellectual disability.

Asterisk indicates statistical significance.

**Table 4:** Spearman Correlation of mean difference between pre and post scores and participant VR Sensory Room usage hours.

Spearman correlation	Usage hours for evenness VR sensory room	
Glasgow Depression Scale (GDS-LD)	R (p)	0.078 (0.68)
Glasgow Anxiety Scale (GAS-ID)	R (p)	0.056 (0.77)
Personal Wellbeing Index (PWI)	R (p)	− 0.21 (0.32)
Adolescent Adult Sensory Profile (AASP)—Low Registration	R (p)	0.38 (0.037*)
Adolescent Adult Sensory Profile (AASP)—Sensation Seeking	R (p)	− 0.058 (0.76)
Adolescent Adult Sensory Profile (AASP)—Sensory Sensitivity	R (p)	0.44 (0.016*)
Adolescent Adult Sensory Profile (AASP)—Sensation Avoiding	R (p)	0.19 (0.32)
Adaptive Behaviour Assessment System (ABAS-3)—Adaptive Composite Score	R (p)	0.081 (0.67)

*R* Spearman Correlation Score.

*Significant at 0.05 level.

*p* significance value.

### No significant change in adaptive behaviour

Following use of the VR Sensory Room, increases were observed for adaptive behaviour, but no statistical significances were found across all ABAS-3 domains as shown in Table 3. No significant correlation was observed between the duration of time spent in the VR Sensory Room and the mean difference in the overall adaptive behaviour between post-test and pre-test (r = 0.081, *p* = 0.67) (See Table 4).

An analysis of the qualitative interviews identified that two of the three staff highlighted that the usage of the VR Sensory Room supported social participation which may, in part, be linked to the construct of adaptive behaviour. One staff member highlighted their perception of social benefit:

*‘It will help them socially interact, because when they're getting quite heightened, they remove themselves from the situation. By putting them in the Sensory Room, instead of removing them from the situation and not returning for the day, they're going in a Sensory Room for five minutes, calming themselves down, re-regulating, and then they're going back into the situation’ (Staff #1).*

## Discussion

The current study is the first to investigate the impact of a VR sensory room intervention on anxiety, depression, personal wellbeing, sensory processing and adaptive behaviour for adults with disability. Assessment included pre- and post-quantitative measures validated for use with adults with disability, and qualitative interviews with a cross section of thirteen participants. Quantitative findings indicated improvements in anxiety and depression from pre to post, with changes in sensory processing, but no significant changes in adaptive behaviour or personal wellbeing. Qualitative findings indicated that participants perceived there was a positive impact on anxiety as well as enhanced social participation.

Positive changes observed in anxiety are promising. This is the first study to examine the impact of a VR Sensory Room on anxiety so there are limited studies to compare to. A pervious study by Harrison et al.^52^ measured the impact of a VR relaxation intervention on the anxiety of adult female athletes with measures of physiology such as heart rate (HR) and kinematics. The VR relaxation intervention comprised a four minute passive audio-visual experience of a camp fire and was found to significantly reduce anxiety, including a lower HR. The VR relaxation intervention was similar to Evenness in that there were no particular demands of participants. However, Harrison et al.’s VR intervention was not interactive and did not comprise the same sensory experiences, nor was the participant population similar.

In the present study, specific improvements were noted in the physiological aspects of anxiety as measured by the GAS-ID including a person’s own perception of their HR or breathing. Previous studies have noted improvements in the physiological or ‘somatic’ aspects of anxiety following immersive VR usage. Linares-Chomorro et al.^53^ observed this with health professionals, although participants did not have disabilities. The present findings may be similar to physiological findings observed by Harrison and colleagues although caution must be applied because the present study did not directly measure physiology.

Previous studies of physical sensory rooms have reported reductions in anxiety in similar populations to the present study^1^. It is possible that the Evenness VR Sensory Room was able to successfully replicate a physical sensory room as intended, with the same observed reductions in anxiety, but in a more accessible virtual format and at a lower cost. More research is needed to explore this finding.

The present study also observed significant reductions in depression for participants who met the threshold that indicated depression prior to the study, with no significant findings in autism or ID subgroups. Very few studies have targeted depression using VR^54^, though promising results were reported with depression severity^55^ and major depression^56^ for people without disability. Studies differed in relation to intervention used, outcome measurement and participant group, with Shah et al. excluding participants with intellectual disability. In many studies targeting anxiety and depression using VR, people with disabilities such as autism and intellectual disability were excluded, despite the known co-morbidity and thus pressing need for intervention.

Correlational analysis indicated no relationship between change scores in anxiety, depression, and amount of VR usage. It is very likely that greater participant numbers would be needed to measure this with more statistical power, or that the ‘right’ amount of usage to effect the changes observed in anxiety and depression is unique to each person.

Changes were also noted in sensory processing in the low registration (increase in scores) and sensory seeking (decrease in scores) quadrants for those with ID and the sensory sensitivity quadrant for autistic participants from pre to post use. Significant correlations observed in sensory sensitivity and low registration scores and VR usage indicated that increased time spent using the VR sensory room may have mediated the changes in sensory processing, however further analysis would be needed to confirm directionality. These findings highlight the sensory processing complexities and differences present in autistic people^2^ and those with and without co-occurring ID^57^. Improvements in sensory processing were reported in the sole study of children with Down syndrome using VR technology to implement a sensory-based intervention on a Wii platform^36^. However, Wuang et al.’s study differs from the present study with regard to type of VR platform, age, disability type and measure of sensory processing. No studies were located which explored the impact of a VR sensory intervention on sensory processing in adults with disabilities.

There were no significant improvements observed in personal wellbeing and adaptive behaviour (encompassing daily living skills such as home living, leisure and self-care) and no significant correlations observed with score changes and VR usage. Very few previous studies measured personal wellbeing and adaptive behaviour changes in response to VR intervention, and only one for people with disability. A small study by Tam, Man^58^ reported positive impact of VR in relation to daily living skills, although findings were not statistically significant and used different outcome measures to the present study. No other published studies were found evaluating the impact of a VR sensory intervention on personal wellbeing or daily living skills.

The findings have implications for both the academic and practitioner community. VR Sensory room participation is presented as a potential tool for improving the immediate emotional wellbeing and sensory processing of vulnerable community members. The length of participation appears to offer minimal impact on outcomes. It could be hypothesised that a VR sensory room may impact anxiety, depression and sensory processing in the short term. Shorter, more regular, assessments of outcomes throughout intervention may provide greater insight into the causal relationships between these constructs, for example, does a change in sensory processing reduce anxiety and depression, or vice versa? Similarly, although not witnessed in the short-term, it is possible that changes in personal wellbeing and adaptive behaviour may require longer term exposure to the VR sensory room, via a ‘theory of change’ framework, where they are cast as medium term outcomes following improvements in anxiety, depression and sensory processing. Further studies are required with a larger number of participants and varying data collection points to test these hypotheses and further inform theory and practice.

### Limitations

This study presents a preliminary investigation into Evenness, and thus there are a number of limitations which warrant caution in interpreting results. First, the sample size was small, with varying ages and disabilities observed in the participants. Sampling of participants was limited to those available and willing to take part at the time and this may present a possible selection bias which may limit the strength and generalizability of the present findings. Second, participants were not given specific direction on the frequency or duration of Evenness usage, rather usage was directed by the user. Third, the design of this study did not include a control group and as such, researchers cannot firmly conclude that benefits observed were as a result of Evenness usage.

### Future directions

Preliminary findings are promising, but future studies would be needed to confirm results observed. Future studies evaluating Evenness could comprise a larger number of participants, a matched control group with randomization to avoid selection bias (such as randomized controlled trial design) and clear guidance on optimal frequency and duration of usage. Future robust studies could also explore factors which may influence VR uptake usage and outcomes such as age, gender and disability type.

## Conclusion

The current results provide preliminary indications of the benefits of a VR sensory room, and insight into who may benefit the most from this intervention. Further research is needed which adopts larger sample sizes, comparator groups, and a study of longer-term outcomes. It is hoped that the current findings may serve as a catalyst for further consideration of the implementation and evaluation of VR sensory rooms for adults with disability to identify how best to bolster wellbeing and community participation.

## Data Availability

The datasets used and/or analysed during the current study available from the corresponding author on reasonable request.
